# Meat Quality, Intestinal Microbiology and Serum Biochemical Parameters of Broilers Fed Different Levels of Green Tea (*Camellia sinensis*) and Mulberry (*Morus alba*) Leaves Powder

**DOI:** 10.1002/vms3.70213

**Published:** 2025-01-16

**Authors:** Fatemeh Aziz‐Aliabadi, Hadi Noruzi, Ahmad Hassanabadi

**Affiliations:** ^1^ Department of Animal Science, Faculty of Agriculture Ferdowsi University of Mashhad Mashhad Iran

**Keywords:** broilers, green tea, intestinal microbiology, meat quality, mulberry leaf, performance, serum biochemical parameters

## Abstract

**Background:**

Today, customers pay more attention to the feed composition and carcasses of poultry, and the interest in using natural and safe compounds such as medicinal plants and their extracts in animal feed is increasing.

**Objectives:**

The present experiment was conducted to assess the effect of green tea (*Camellia sinensis*) and mulberry (*Morus alba*) leaves powder on the meat quality, intestinal microbiology and serum biochemical parameters in broilers.

**Methods:**

The experiment was conducted with 648 one‐day‐old Ross 308 broiler male chicks with a factorial arrangement including three levels of green tea powder (GTP) and three levels of mulberry leaf powder (MLP), with nine treatments and six replications in a completely randomized design for 42 days. Treatments included: (1) no GTP + no MLP (control), (2) 1% GTP + no MLP, (3) 2% GTP + no MLP, (4) no GTP + 1% MLP, (5) 1% GTP + 1% MLP, (6) 2% GTP + 1% MLP, (7) no GTP + 2% MLP, (8) 1% GTP + 2% MLP and (9) 2% GTP + 2% MLP.

**Results:**

The results showed that the lowest lightness (L*), drip loss and total cholesterol levels, and the highest *Lactobacillus* population were observed in treatments: 1% GTP + no MLP, 2% GTP + no MLP, 1% GTP + 1% MLP, 2% GTP + 1% MLP, no GTP + 2% MLP, 1% GTP + 2% MLP and 2% GTP + 2% MLP (*p* < 0.05). The groups receiving 1% GTP + 1% MLP, 2% GTP + 1% MLP, no GTP + 2% MLP, 1% GTP + 2% MLP and 2% GTP + 2% MLP had the highest pH 24 h (*p* < 0.05). The chickens fed with 1% and 2% GTP showed lower low‐density lipoprotein cholesterol (LDL) and malondialdehyde (MAD) levels (*p* < 0.05).

**Conclusions:**

The results showed that using the GTP and MLP in the diet of broilers could improve meat quality traits and beneficial ileal bacteria populations and reduce serum lipid and MDA levels.

## Graphical abstract

•648 one‐day‐old Ross 308 broiler chickens were reared for 42 days with nine experimental diets.

•The use of green tea and mulberry leaves powder improved the meat quality and the ileum beneficial bacteria population and reduced the serum cholesterol, low‐density lipoprotein and malondialdehyde concentrations.

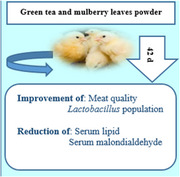

## Introduction

1

Nowadays, there are various types of antibiotic substitutes used currently, among which natural substances and medicinal plants with great physiological activity are getting consideration by researchers (Hernandez et al. 2004). Green tea (*Camellia sinensis*) is considered a feasible source of suitable nutrients, antibiotic substitution and natural antioxidants for animals (Cyril and Jozef 2017). Mulberry (*Morus alba*) leaves are rich in crude protein with an excellent amino acid profile, carbohydrates, fats, minerals, fibres and metabolizable energy (Sanchez 2002). It has been reported that plants rich in flavonoids, such as green tea, mulberry leaves and thyme, have an antibacterial effect. These herbs can enhance the growth performance and immune response (Cook and Samman 1996). Modern broiler strains are distinguished by fast‐growing, high marketing weight and high feed intake to meet their requirements. However, these properties aim for body weight gain and feed conversion ratio (FCR) without considering some unavoidable issues such as metabolic disorders and low meat quality (Bahareldin et al. 2018). Several studies have shown that green tea reduces the production of free radicals and lowers blood cholesterol levels (Muramatsu, Fukuyo, and Hara 1986) and cancer incidence (Mukhtar and Ahmad 1999). Nowadays, the physiological maintenance and gut morphology improvement is the main focus of gut safety for human and animal nutrition researchers. Animals with healthy gastrointestinal tracts are less affected by biotoxins, toxic microbiomes or microbial metabolites with other adverse environmental hazards. Due to the antibacterial, antifungal, antioxidant, antiparasitic and antiviral effects of phytochemicals, they can reduce microbial pathogen populations and protect the intestinal microvilli (Hashemipour et al. 2013). It has been stated that the phenolic compounds (such as tannin) in green tea leaves can disrupt the utilization of protein and other nutrients and interfere with bird performance (Mahlake et al. 2021). In addition, due to the presence of abundant crude fibre and tannin in mulberry leaf, high dietary levels of it can also negatively affect poultry performance and health (Al‐kirshi et al. 2010). As a result, the presence of these anti‐nutritional substances in mulberry leaf will lead to restrictions on their consumption in poultry diets (Srivastava et al. 2006). According to our research, few studies have investigated the combined effect of green tea powder (GTP) and mulberry leaves powder (MLP) in broilers. Considering the significant effects of the GTP and MLP on the broiler's performance, immunity and intestinal morphology in the first part of our study (Aziz‐Aliabadi, Noruzi, and Hassanabadi 2023), the authors decided to carry out this study to determine the most optimal amounts of GTP and MLP in the diets, which positively affect meat quality, intestinal microbiology and serum biochemical parameters in broiler chickens.

## Materials and Methods

2

### Preparation and Analysis of Green Tea and Mulberry Leaves Powder

2.1

Dry green tea and mulberry leaves were provided from a local market in Rasht, Iran. All samples were ground by a Grinder (Kinematica AG, Malters, Switzerland) to a particle size of 2 mm. These additives were added on top of the diets. The percentage of dry matter (91.46 and 91.77), crude protein (19.34 and 16.80), crude fibre (13.29 and 14.95), crude fat (2.06 and 3.12) and ash (5.65 and 10.12) of GTP and MLP were determined, respectively (AOAC 2005).

### Birds, Diets, and Management

2.2

All procedures were approved by The Animal Care and Use Committee of the Ferdowsi University of Mashhad, Iran.

To carry out this experiment, 648 one‐day‐old Ross 308 broiler male chicks were acquired from a commercial hatchery and reared in pens for 42 days. The experimental diets (3 × 3 with six replicates) were fed to the chickens during the starter (1–10 days of age), grower (11–24 days of age) and finisher (25–42 days of age) phases (Table 1) and included the following: (1) no GTP + no MLP (control), (2) 1% GTP + no MLP, (3) 2% GTP + no MLP, (4) no GTP + 1% MLP, (5) 1% GTP + 1% MLP, (6) 2% GTP + 1% MLP, (7) no GTP + 2% MLP, (8) 1% GTP + 2% MLP and (9) 2% GTP + 2% MLP. Each treatment contained six replicates with 12 birds each. See the previous study of the authors to observe the composition of the diets (Aziz‐Aliabadi, Noruzi, and Hassanabadi 2023). All experimental mash diets were formulated to meet nutrient requirements of Ross 308 (Aviagen 2014), and herbal powder was added to basal diets (on top). The house temperature was constant at 32°C during the first 3 days of rearing and decreased by 3°C every week until it remained constant at 21°C. Relative humidity was held between 50% and 60% during the experimental period. The lighting programme was considered as 18 h light and 6 h dark during the study. Feed and water were presented ad libitum throughout the experiment (Noruzi et al. 2022).

**TABLE 1 vms370213-tbl-0001:** Effects of different levels of green tea and mulberry leaves powder on breast meat quality parameters of broilers at 42 days of age.

Treatments								
	Lightness	Redness	Yellowness	pH		Drip loss	Cooking loss	Shear force
Main effects	(L^*^)	(a^*^)	(b^*^)	45 min	24 h	(%)	(%)	(N)
GTP								
0	48.52^a^	9.02	18.30	6.38	5.80^b^	4.54^a^	19.92	33.26
1	47.09^c^	8.89	18.78	6.49	6.09^a^	4.21^b^	19.70	33.14
2	47.53^b^	8.95	18.56	6.47	6.02^a^	4.19^b^	19.77	33.21
*p* value	0.003	0.83	0.08	0.52	0.01	0.005	0.06	0.66
SEM	0.115	0.241	0.145	0.076	0.061	0.080	0.064	0.099
MLP (%)								
0	48.06^a^	9.11	18.39	6.46	5.84^b^	4.44	19.82	33.23
1	47.51^b^	9.02	18.67	6.43	6.01^a^	4.31	19.74	33.26
2	47.57^b^	8.74	18.57	6.45	6.05^a^	4.20	19.83	33.12
*p* value	0.02	0.23	0.39	0.94	0.04	0.13	0.59	0.55
SEM	0.119	0.156	0.147	0.079	0.061	0.080	0.064	0.093
Interaction effects								
no GTP + no MLP	49.21^a^	8.85	17.89	6.31	5.56^b^	4.94^a^	20.04	33.51
1% GTP + no MLP	47.37^cd^	9.13	18.61	6.48	5.99^ab^	4.22^b^	19.61	33.20
%2 GTP + no MLP	47.60^bcd^	9.34	18.68	6.59	5.98^ab^	4.15^b^	19.81	32.98
no GTP + 1% MLP	48.35^ab^	9.24	18.62	6.38	5.83^ab^	4.51^ab^	19.77	33.26
1% GTP + 1% MLP	46.78^d^	8.88	18.97	6.46	6.24^a^	4.18^b^	19.70	33.21
2% GTP + 1% MLP	47.42^cd^	8.93	18.42	6.43	6.15^a^	4.25^b^	19.76	33.31
no GTP + 2% MLP	48.01^bc^	8.98	18.39	6.43	6.00^a^	4.19^b^	19.94	33.00
1% GTP + 2% MLP	47.14^cd^	8.65	18.77	6.54	6.05^a^	4.24^b^	19.79	33.02
2% GTP + 2% MLP	47.56b^cd^	8.59	18.56	6.39	6.11^a^	4.18^b^	19.75	33.34
*p* value	0.04	0.48	0.43	0.78	0.04	0.04	0.46	0.13
SEM	0.199	0.271	0.250	0.132	0.127	0.139	0.111	0.161

*Note*: 0, 1, 2: different levels of GTP and MLP (%).

Abbreviations: GTP, green tea powder; MLP, mulberry leaf powder; SEM, standard error of the mean.

^a–d^Means within a column without a common superscript significantly differ (*p* < 0.05).

### Meat Quality

2.3

At 42 days of age, two birds, with an average body weight of each pen, were selected and euthanized by cervical dislocation. The right breast muscle was removed and kept at 4°C to measure meat quality.

#### Meat Colour

2.3.1

A CR410 chromameter (Konica Minolta Sensing Inc., Japan) was used to evaluate muscle colour traits (lightness L*, redness a* and yellowness b*) 24 h after death (W. Zhang et al. 2011).

#### pH

2.3.2

A WTW pH 330i pH Meter (Germany) was used to assess the pH of the muscle at 45 min and 24 h post‐mortem (L. Zhang et al. 2009).

#### Drip Loss

2.3.3

The drip loss of meat samples was determined 24 h after death (C. Zhang et al. 2017). A piece of muscle sample (2 × 2 × 5 cm) was weighed and put in a plastic pocket and kept suspended at 4°C. After 24 h, the samples were wiped and reweighed. The drip loss (%) of the meat was measured based on the following formula:

$$\mathrm{Drip}\mathrm{loss}\left(\%\right)=\left[\left(\mathrm{initial}\mathrm{weight}-\mathrm{final}\mathrm{weight}\right)/\mathrm{initial}\mathrm{weight}\right]\times 100$$



#### Cooking Loss and Shear Force

2.3.4

Cooking loss and shear force were evaluated according to C. Zhang et al. (2015). Briefly, a piece of meat (2 × 2 × 5 cm) was weighed and then put into a zip‐sealed plastic bag and cooked in a water bath at 85°C. The samples were removed after 30 min of cooking and put at 4°C. Then, samples were blotted dry on filter paper and reweighed. Cooking loss (%) was determined according to the following formula:

$$\mathrm{Cooking}\mathrm{loss}\left(\%\right)=\left[\left(\mathrm{initial}\mathrm{weight}-\mathrm{cooked}\mathrm{final}\mathrm{weight}\right)/\mathrm{initial}\mathrm{weight}\right]\times 100$$



Then, the cooked samples were analysed for shear force evaluation using a Texture Analyser (TA.XT plus, Stable Micro Systems, UK) according to the procedure described by L. Zhang et al. (2014).

### Intestinal Microbiology

2.4

Two chickens from each replicate were randomly chosen and slaughtered, and then 1 g of digesta was removed from the ileum of each bird. It was diluted and homogenized with 9 mL of physiological serum (85%) to investigate the intestinal microbial flora at 42 days of age. The samples were used for counting bacteria, including *Lactobacillus* and *Coliforms*, which were cultured using MRS Agar and MacConkey Agar, and incubated at 37°C for 72 and 48 h (McCartney, Wenzhi, and Tannock 1996). The total number of colonies was expressed as log 10 cfu/g.

### Determination of Serum Biochemical and Oxidative Indices

2.5

At 42 days of age, blood samples were obtained via the wing vein from two birds in each pen. Blood samples were centrifuged (2000 × *g* for 10 min), and serum was separated and then stored at −20°C until assayed to measure serum lipid profile and malondialdehyde (MDA). The serum concentration of triglycerides (Bucolo and David 1973), total cholesterol (Zak et al. 1954) and high‐density lipoprotein cholesterol (HDL) (Naito and Kaplan 1984) was determined by appropriate laboratory kits (Pars Azmoon, Tehran, Iran). The concentration of very low‐density lipoprotein cholesterol (VLDL) was determined by dividing triglyceride levels by 5, and low‐density lipoprotein cholesterol (LDL) was calculated by the traditional Friedewald equations (Friedewald, Levy, and Fredrickson 1972). The activity of serum MDA was assessed by appropriate kits (Cayman Chemical, Ann Arbor, MI, USA) according to the company's manual and read by spectrophotometer (UNICO 2100, UV–Visible).

### Statistical Analyses

2.6

This experiment was carried out according to a completely randomized design in a factorial arrangement of 3 × 3 with three levels of GTP and three levels of the MLP by the GLM procedure of SAS 9.4 (Statistical Analyses System (SAS) 2012) software. Results are shown as means ± standard error of the mean (SEM). All data were normalized by the Shapiro–Wilk test. The significance of treatment differences was analysed using ANOVA. Tukey's test was used to compare the means of treatments (*p* < 0.05).

## Results

3

### Meat Quality

3.1

The effects of different levels of GTP and MLP on meat quality at 42 days of age are presented in Table 1. The lowest L* value (*p* < 0.05) was observed in the birds consuming 1% GTP as compared to the other treatments (control and 2% GTP). Regarding the main effects of MLP, the groups receiving 1% and 2% MLP showed lower L* as compared to the control (*p* < 0.05). Regarding the interaction effects of GTP and MLP, the lowest L* was observed in treatments: 1% GTP + no MLP, 2% GTP + no MLP, 1% GTP + 1% MLP, 2% GTP + 1% MLP, no GTP + 2% MLP, 1% GTP + 2% MLP and 2% GTP + 2% MLP, which were significantly different as compared to the control (*p* < 0.05). The highest pH 24 h value was perceived in the birds receiving 1% and 2% GTP or 1% and 2% MLP as compared to the control group (*p* < 0.05). The treatments containing 1% GTP + 1% MLP, 2% GTP + 1% MLP, no GTP + 2% MLP, 1% GTP + 2% MLP and 2% GTP + 2% MLP showed the highest pH 24 h (*p* < 0.05). Birds in the 1% and 2% GTP groups showed lower (*p* < 0.05) drip loss at 24 h compared with the control. Concerning the interaction effects of GTP and MLP, the lowest drip loss was observed in the groups containing 1% GTP + no MLP, 2% GTP + no MLP, 1% GTP + 1% MLP, 2% GTP + 1% MLP, no GTP + 2% MLP, 1% GTP + 2% MLP and 2% GTP + 2% MLP (*p* < 0.05). No significant differences with respect to pH 45 min, a* value, b* value, cooking loss and shear force were detected among the experimental treatments (*p* > 0.05).

### Intestinal Microbiology

3.2

The results of counting *Lactobacillus* and *Coliforms* in the ileum of broilers are illustrated in Table 2. The main effects of GTP on the *Lactobacillus* population showed a significant difference (*p* < 0.05), so the groups consuming 1% and 2% GTP showed an increase in the number of *Lactobacillus*. The highest *Lactobacillus* population was observed in treatments: 1% GTP + no MLP, 2% GTP + no MLP, 1% GTP + 1% MLP, 2% GTP + 1% MLP, no GTP + 2% MLP, 1% GTP + 2% MLP and 2% GTP + 2% MLP, which were significantly different compared to the control group (*p* < 0.05). The population of *Coliforms* was not affected by the experimental treatments (*p* > 0.05).

**TABLE 2 vms370213-tbl-0002:** Effects of different levels of green tea and mulberry leaves powder on ileum microflora at 42 days of age (mean log10 cfu/g).

Treatments		
Main effects	*Lactobacillus*	*Coliforms*
GTP (%)		
0	5.36^b^	5.12
1	5.58^a^	5.14
2	5.56^a^	5.04
*p* value	0.005	0.19
SEM	0.034	0.041
MLP (%)		
0	5.44	5.13
1	5.53	5.08
2	5.52	5.10
*p* value	0.17	0.68
SEM	0.039	0.041
Interaction effects		
no GTP + no MLP	5.17^b^	5.19
1% GTP + no MLP	5.54^a^	5.15
2% GTP + no MLP	5.60^a^	5.04
no GTP + 1% MLP	5.43^ab^	5.12
1% GTP + 1% MLP	5.63^a^	5.12
2% GTP + 1% MLP	5.54^a^	5.01
no GTP + 2% MLP	5.49^a^	5.07
1% GTP + 2% MLP	5.56^a^	5.13
2% GTP + 2% MLP	5.52^a^	5.06
*p* value	0.04	0.89
SEM	0.068	0.071

*Note*: 0, 1, 2: different levels of GTP and MLP (%).

Abbreviations: GTP, green tea powder; MLP, mulberry leaf powder; SEM, standard error of the mean.

^a,b^Means within a column without a common superscript significantly differ (*p* < 0.05).

### Serum Biochemical and Oxidative Indices

3.3

The main and combined effects of GTP and MLP on the serum biochemical and oxidative parameters are highlighted in Table 3. Regarding the main effect of GTP, the groups fed with 1% and 2% GTP had lower total cholesterol compared to the control (*p* < 0.05). Concerning the main effect of MLP, the lowest total cholesterol level was observed in the broilers receiving 2% MLP compared to the control group (*p* < 0.05). The birds feeding with 1% and 2% GTP showed lower LDL and MDA compared to the control (*p* < 0.05). Regarding the interaction effects of GTP and MLP, treatments containing 1% GTP + no MLP, 2% GTP + no MLP, 1% GTP + 1% MLP, 2% GTP + 1% MLP, no GTP + 2% MLP, 1% GTP + 2% MLP and 2% GTP + 2% MLP showed lower total cholesterol levels compared to the control (*p* < 0.05).

**TABLE 3 vms370213-tbl-0003:** Effects of different levels of green tea and mulberry leaves powder on the serum biochemical and oxidative indices of broilers at 42 days of age.

Treatments					
Main effect	Total cholesterol (mg/dL)	HDL (mg/dL)	LDL (mg/dL)	VLDL (mg/dL)	MDA (nmol/mL)
GTP (%)					
0	186.00^a^	70.83	96.64^a^	17.51	9.58^a^
1	173.56^b^	70.56	86.12^b^	16.99	8.64^b^
2	173.61^b^	69.78	87.49^b^	17.09	8.42^b^
*p* value	< 0.0001	0.40	0.02	0.15	< 0.0001
SEM	1.792	1.132	2.838	0.199	0.157
MLP (%)					
0	182.28^a^	71.22	92.96	17.54	9.18
1	179.22^a^	71.94	90.55	17.19	8.73
2	171.67^b^	69.02	86.73	16.88	8.72
*p* value	0.004	0.10	0.29	0.07	0.08
SEM	1.815	1.154	2.758	0.195	0.157
Interaction effects					
no GTP + no MLP	191.32^a^	70.67	100.90	17.71	10.07
1% GTP + no MLP	179.50^bc^	72.83	89.46	17.58	8.94
2% GTP + no MLP	176.00^bc^	70.17	88.52	17.32	8.53
no GTP + 1% MLP	192.34^a^	72.83	99.82	17.93	9.32
1% GTP + 1% MLP	176.36^bc^	71.85	88.05	16.45	8.52
2% GTP + 1% MLP	169.02^bc^	68.16	83.78	17.18	8.36
no GTP + 2% MLP	174.37^bc^	69.03	89.18	16.92	9.35
1% GTP + 2% MLP	164.85^c^	67.12	80.87	16.96	8.46
2% GTP + 2% MLP	175.83^bc^	68.21	90.14	16.77	8.37
*p* value	0.003	0.62	0.45	0.19	0.83
SEM	3.103	1.998	4.916	0.364	0.272

*Note*: 0, 1, 2: different levels of GTP and MLP (%).

Abbreviations: GTP, green tea powder; HDL, high‐density lipoprotein cholesterol; LDL, low‐density lipoprotein cholesterol; MDA, malondialdehyde; MLP, mulberry leaf powder; SEM, standard error of the mean; VLDL, very low‐density lipoprotein cholesterol.

^a–c^ Means within a column without a common superscript significantly differ (*p* < 0.05).

## Discussion

4

Due to high contents of polyphenolic catechins (epigallocatechin, epigallocatechin gallate and epicatechin gallate) in the green tea, it is considered a source of antioxidants. It has been stated that the green tea polyphenols work in the plasma membrane alongside proteins and phospholipids, and these components regulate signal transition routes, transcription agents, methylation of DNA, the function of mitochondria and autophagy (Kim, Quon, and Kim 2014). Mulberry leaf is a vital component in some traditional medicinal formulations, and it has high nutritional value and antioxidant activity (Krol et al. 2016). Our previous study showed that there was no considerable difference among the birds in terms of feed intake. However, the consumption of various amounts of GTP and MLP enhanced the weight gain, and these changes led to ameliorated FCR (Aziz‐Aliabadi, Noruzi, and Hassanabadi 2023).

The presence of polyphenols in medicinal plants boosts meat quality due to their antioxidant properties (Botsoglou et al. 2002). In the present study, diets supplemented with different levels of GTP and MLP decreased the value of L* in breast meat. It has been reported that GTP and MLP may increase the antioxidant capacity of cells by preventing cell sap extravasation and resulting in decreasing light reflection and L* value (Niu et al. 2017). On the other hand, some researchers stated another reason for the decrease in L* value. Xie et al. (2022) reported that the antioxidation of olive leaf extract prevented the autoxidation of haem and led to lowering the breast muscle lightness and enhancing the breast muscle redness. Jang et al. (2008) stated that a dietary medicinal herb extract mix consisting of mulberry leaf, Japanese honeysuckle and goldthread resulted in lower L* values of breast meat. They suggested that lower L* values may be related to the high pH values. White muscle fibres of chicken breast contain low myoglobin. In addition, pH is mainly an important criterion to evaluate the goodness or badness of meat. The amount and velocity of pH reduction in the post‐mortem muscle could directly affect the tint, tenderness, taste, water preservation and storage time of the meat (Turgut, Is¸ıkçı, and Soyer 2017). In the present study, although the pH 45 min did not affect treatments, the pH 24 h remarkably mounted, showing that the pH of the broiler meat diminished more slowly 24 h post‐mortem via dietary GTP and MLP supplementations. Wu et al. (2014) observed a decrease in meat drip loss due to using GTP in ducks. They stated that due to its antioxidant properties, green tea reduces the oxidation of lipids and increases the integrity of the cell wall, and thus improves the water‐holding capacity in meat. In the present study, diets containing GTP and MLP substantially decreased drip loss. In accordance with these results, Geng et al. (2024) observed that different levels (low, moderate and high) of mulberry leaves affect the meat pH and drip loss.

It has been reported that the antimicrobial attributes of herbals can ameliorate the microbial population and enteric well‐being of the digestive tract of birds by decreasing the pathogenic bacteria colonization (Yadav et al. 2016; Jin, Dersjant‐Li, and Giannenas 2020). Axling et al. (2012) reported that GTP, in combination with a single strain of *Lactobacillus plantarum*, was able to improve the growth of *Lactobacillus* in the intestine of mice. Chen, Ni, and Li (2019) demonstrated that GTP and MLP could significantly affect the gut microbiota of chickens by altering their composition. They stated that GTP significantly affected the bacterial diversity in the broiler's gut by increasing the prevalence of *Proteobacteria*. In addition, in the mentioned study, the difference in the core microbiota at the family and genus level was reported between the feed supplemented with MLP and the non‐supplemented diets (Chen, Ni, and Li 2019). It has been reported that the use of mulberry leaf extract increased the number of beneficial bacteria, such as *Bifidobacterium* and *Lactobacillus*, while decreased the presence of pathogenic *Escherichia coli* in the piglets’ colon (Song et al. 2023). Aziz‐Aliabadi, Noruzi, and Hassanabadi (2023) attributed the improvement of villus height to the presence of polyphenols in the green tea, which effectively prevent the proliferation of pathogenic bacteria and improve the intestinal health. Sigolo et al. (2021) reported that plant extracts reduced *Coliforms* and aerobic bacteria in the ileum. In our study, using GTP and MLP could increase the population of *Lactobacillus* bacteria. High *Lactobacillus* populations can characterize the acidification of the digestive tract and stimulate the proliferation of beneficial microbiota, such as the lactic acid bacteria. By producing short‐chain fatty acids, this kind of bacteria enhances intestinal acidity and inhibits the growth of undesirable bacteria like *Salmonella* and *Coliforms* (Giannenas et al. 2018). In addition, it has been stated that the dietary inclusion of GTP increased the number of *Lactobacillus* spp. and reduced the number of pathogenic bacteria such as *Clostridium* spp. (Thomas et al. 2022).

It has been stated that green tea polyphenols decreased serum cholesterol by reducing the absorption of dietary and biliary cholesterol and increasing its fecal excretion (Koo and Noh 2007), or by preventing cholesterol synthesis in the liver through inactivation of 3‐hydroxy‐3‐methylglutaryl coenzyme A reductase and activation of adenosine monophosphate kinase (Yoshino et al. 1994; Singh, Banerjee, and Porter 2009). Khalaji et al. (2011) reported a considerable decrease in total cholesterol at 500 mg/kg of green tea extract. In the present study, treatments containing 1% GTP + no MLP, 2% GTP + no MLP, 1% GTP + 1% MLP, 2% GTP + 1% MLP, no GTP + 2% MLP, 1% GTP + 2% MLP and 2% GTP + 2% MLP showed the lowest amount of total cholesterol compared to the control group. In agreement with our results, the helpful role of herbals on the serum cholesterol concentration has been reported (Abd El‐Hack et al. 2020). Qin et al. (2023) stated that using mulberry leaf extract reduced serum total cholesterol. Adeyemi et al. (2022) reported that the broilers receiving *Solanum aethiopicum* and *Solanecio biafrae* leaves had lower whole cholesterol compared to the control group. MDA is one of the products of lipid peroxidation, and its content in the serum shows the extent of lipid peroxidation damage (Chauhan et al. 2021). The present study showed that dietary supplementation of GTP reduced serum MDA content. Antioxidant‐rich products as dietary additives stimulate the physiological antioxidant defence and reduce oxidative stress (Sadeghi et al. 2013). A meta‐analysis of Geng et al. (2024) showed that low, medium and high levels of mulberry leaf reduced the MDA levels considerably. Sahin et al. (2010) attributed the antioxidant properties of green tea to its polyphenol content, which destroys free radicals, stabilizes the cell wall and prevents lipid peroxidation. On the other hand, some researchers stated that the reduction of liver and plasma MDA levels could be due to: (1) catechin inhibitory activity of superoxide and hydroxyl radicals, (2) chelation with metal ions and formation of inactive complexes and (3) stimulation of the synthesis of endogenous antioxidant enzymes in cells (Kara et al. 2016).

## Conclusions

5

The use of different levels of GTP and MLP reduced the lightness, drip loss and serum total cholesterol and increased the ileum *Lactobacillus* population and breast muscle pH 24. In addition, using 1% green tea leaf powder in the diet considerably reduced the LDL and MDA level in the serum.

## Author Contributions


**Fatemeh Aziz‐Aliabadi**: conceptualization, data curation, formal analysis, funding acquisition, investigation, methodology, project administration, resources, software, writing–original draft, writing–review and editing. **Hadi Noruzi**: conceptualization, data curation, formal analysis, funding acquisition, investigation, methodology, project administration, resources, software, writing–original draft, writing–review and editing. **Ahmad Hassanabadi**: conceptualization, investigation, methodology, supervision, writing–review and editing.

## Ethics Statement

All procedures were approved by the Animal Care and Use Committee of the Ferdowsi University of Mashhad, Mashhad, Iran.

## Conflicts of Interest

The authors declare no conflicts of interest.

### Peer Review

The peer review history for this article is available at https://publons.com/publon/10.1002/vms3.70213.

## References

## Data Availability

The data that support the findings of this study are available from the corresponding author upon reasonable request.
